# Updated Clinical Classification and Hemodynamic Definitions of Pulmonary Hypertension and Its Clinical Implications

**DOI:** 10.3390/jcdd11030078

**Published:** 2024-02-27

**Authors:** Mithum Kularatne, Christian Gerges, Mitja Jevnikar, Marc Humbert, David Montani

**Affiliations:** 1 Division of Respiratory Medicine, Department of Medicine, University of Calgary, Calgary, AB T2N 1N4, Canada; 2 Department of Internal Medicine II, Division of Cardiology, Medical University of Vienna, 1090 Vienna, Austria; 3 Assistance Publique-Hôpitaux de Paris (AP-HP), Department of Respiratory and Intensive Care Medicine, Pulmonary Hypertension National Referral Center, DMU 5 Thorinno, Hôpital Bicêtre, 94270 Le Kremlin-Bicêtre, France; 4 School of Medicine, Université Paris-Saclay, 94270 Le Kremlin-Bicêtre, France; 5 INSERM UMR_S 999 “Pulmonary Hypertension: Pathophysiology and Novel Therapies”, Hôpital Marie Lannelongue, 92350 Le Plessis-Robinson, France

**Keywords:** pulmonary hypertension, right heart catheterization, pulmonary vascular disease

## Abstract

Pulmonary hypertension (PH) refers to a pathologic elevation of the mean pulmonary artery pressure (mPAP) and is associated with increased morbidity and mortality in a wide range of medical conditions. These conditions are classified according to similarities in pathophysiology and management in addition to their invasive hemodynamic profiles. The 2022 ESC/ERS guidelines for the diagnosis and treatment of pulmonary hypertension present the newest clinical classification system and includes significant updates to the hemodynamic definitions. Pulmonary hypertension is now hemodynamically defined as an mPAP > 20 mmHg, reduced from the previous threshold of ≥25 mmHg, due to important insights from both normative and prognostic data. Pulmonary vascular resistance has been extended into the definition of pre-capillary pulmonary hypertension, with an updated threshold of >2 Wood Units (WU), to help differentiate pulmonary vascular disease from other causes of increased mPAP. Exercise pulmonary hypertension has been reintroduced into the hemodynamic definitions and is defined by an mPAP/cardiac output slope of >3 mmHg/L/min between rest and exercise. While these new hemodynamic thresholds will have a significant impact on the diagnosis of pulmonary hypertension, no evidence-based treatments are available for patients with mPAP between 21–24 mmHg and/or PVR between 2–3 WU or with exercise PH. This review highlights the evidence underlying these major changes and their implications on the diagnosis and management of patients with pulmonary hypertension.

## 1. Introduction

**Figure 1:**
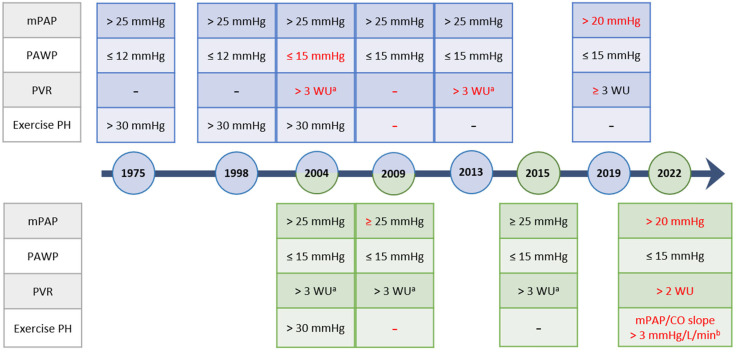
Evolution of the World Symposium (Blue) and ESC/ERS (Green) Hemodynamic Definitions Over Time. Dates are listed based on the year of publication. Updates are highlighted in red. mPAP, mean pulmonary artery pressure; PAWP, pulmonary artery wedge pressure; PVR, pulmonary vascular resistance; PH, pulmonary hypertension; WU, Wood Units. ^a^: applies only to Group 1 PAH, ^b^: mean pulmonary artery pressure to cardiac output slope (mPAP/CO slope) measured between rest and exercise.

Pulmonary hypertension (PH) refers to a group of diseases characterized by a pathologic elevation in the mean pulmonary artery pressure (mPAP). The physiology causing the elevated pulmonary pressures varies significantly based on the underlying etiology including intrinsic pulmonary vasculopathy, changes involving the left heart or pulmonary parenchyma, and chronic thromboembolic disease, among others. Given the large number of potential causes, these diseases are classified using the WHO Clinical Classification which divides the diseases based on their similar underlying mechanisms, presentations, hemodynamics, as well as treatment strategies (Table 1) [1]. Since the publication of the first clinical classification of PH, several advances have been made in understanding the pathophysiologic mechanisms underpinning certain forms of PH. To obtain a diagnosis of PH, regardless of etiology, direct hemodynamic confirmation by right heart catheterization is required. The diagnosis of PH is established based on mPAP but additional hemodynamic findings, including the pulmonary vascular resistance (PVR) and pulmonary arterial wedge pressure (PAWP), are used to further classify the diagnosis (Table 2). Over the past 50 years, as our understanding of the pathologies underlying PH has evolved, the thresholds for these hemodynamic categories have changed, as reflected in the published international guidelines for the diagnosis and management of PH [1,2,3,4,5,6,7,8,9,10] (Figure 1). As the hemodynamic criteria serve as the foundation to the diagnosis of PH, these updates can drastically impact both the diagnosis and the subsequent interventions which patients are offered [1]. This review focuses primarily on the updated hemodynamic definitions in the recent 2022 ESC/ERS Guidelines for the Diagnosis and Treatment of Pulmonary Hypertension and their clinical implications.

## 2. Clinical Classification of Pulmonary Hypertension

The updated clinical classification of PH is based around five categories of diseases organized around similar pathophysiology, hemodynamics, and/or therapeutic management strategies (Table 1). Group 1 pulmonary arterial hypertension (PAH) represents a heterogenous group of conditions, all characterized by progressive pathologic remodeling of the small-calibre pulmonary arteries leading to progressive right ventricular dysfunction and death. The group encompasses idiopathic PAH, heritable PAH, drug and toxin associated PAH, and PAH associated with systemic diseases. Group 2 PH includes diseases of the left heart, such as heart failure with reduced ejection fraction, heart failure with preserved ejection fraction and valvular heart disease. Group 3 PH includes PH due to lung diseases and/or hypoxia. Group 4 pulmonary hypertension is associated with pulmonary artery obstruction such as chronic thromboembolic pulmonary hypertension. Group 5 pulmonary hypertension is due to conditions leading to elevated pressures for unclear and/or multifactorial mechanisms. These groups were retained from prior iterations of the clinical classification, but three main updates and some minor changes were included.

Firstly, idiopathic PAH (within Group 1) was divided based on response to acute vasoreactivity testing results at right heart catheterization. During right heart catheterization, patients are exposed to an agent, typically inhaled nitric oxide, to assess for acute changes in pulmonary hemodynamics. The criteria for a positive response remain unchanged from previous iterations, and is defined as a reduction in the mPAP by ≥10 mmHg to an absolute value of ≤40 mmHg with an unchanged or increased cardiac output [11]. A positive response identifies patients, termed “acute responders”, who may benefit from high dose calcium channel blocker therapy and predicts a favourable long-term outcome [12]. However, while approximately 12% of patients are found to have an acute response at vasoreactivity testing, only around 7% have a persistent clinical and hemodynamic response after at least one year on high dose calcium channel blocker therapy [11]. These longer-term responders are the ones with the more favourable outcome and should be included within this classification. While response to vasoreactivity testing has been included as a subcategory of idiopathic PAH, patients with heritable and drug- or toxin-associated PH may also be acute responders and may benefit from calcium channel blocker therapy, thus testing is also indicated in those subgroups.

The second change is the recategorization of PAH with features of venous/capillary (pulmonary veno-occlusive disease/pulmonary capillary haemangiomatosis (PVOD/PCH) involvement) and persistent PH of the newborn into group 1 PAH. In the prior guidelines, they were provided with their own special subcategorization of 1′ and 1″, respectively.

The third main change is the terminology change for group 3.4 from sleep disordered breathing to the new term hypoventilation syndrome. This change comes from the accumulation of data showing that PH with obstructive sleep apnea is rare when not associated with conditions leading to hypoxemia [13].

Some other minor changes include the movement of PH associated with lymphangioleiomyomatosis (LAM) into group 3 PH associated with chronic lung disease. Recent studies revealed that PH in patients with LAM was correlated to the degree of pulmonary function impairment and hypoxemia, making it more appropriately classified into group 3 PH. Additionally, splenectomy and thyroid disorders have been removed from the classification schema as these conditions are not felt to cause PH, but are rather associated comorbidities. These minor changes are in line with the proceedings of the 6th World Symposium of Pulmonary Hypertension (WSPH) [14].

Overall, while the importance of the clinical classification in defining further management cannot be understated, the changes included in this 2022 ESC/ERS classification update are unlikely to significantly impact clinical practice in contrast to the changes in the hemodynamic definitions.

## 3. Hemodynamic Definition of Pulmonary Hypertension

PH has been defined as mPAP of ≥25 mmHg since the proceedings of the first WSPH in 1975 [2]. However, the mPAP alone is insufficient for adequate discrimination between patients with pulmonary vascular disease from causes such as an increased cardiac output (CO) or increased left ventricular filling pressures [2,9]. As a result, subsequent hemodynamic definitions included thresholds for the PAWP and, more recently, the PVR to help distinguish different causes of the increased mPAP. In the 2015 ESC/ERS Guidelines, pre-capillary PH was defined as also requiring a PAWP of ≤15 mmHg to distinguish it from PH due to left heart disease. While a PVR threshold was not included in the diagnosis of pre-capillary PH, the diagnosis of Group 1 PAH required a threshold of ≥3 Wood units (WU) in the absence of other causes of PH, such as severe parenchymal lung disease [9]. In the 2022 update, major updates from the 2015 iteration were made to the hemodynamic definition reducing the thresholds of PH to an mPAP > 20 mmHg and the PVR threshold to >2 WU to define pre-capillary PH, while the PAWP cut-off of ≤15 mmHg was maintained to distinguish pre-capillary from post-capillary PH. Additionally, exercise induced PH was reintroduced into the hemodynamic classification. Other hemodynamic variables that have been shown to provide important prognostic data, such as pulmonary artery compliance, were not included in the updated definitions [15].

### 3.1. Mean Pulmonary Artery Pressure

The mPAP threshold was established relatively arbitrarily during the first WSPH as ≥25 mmHg, despite evidence at that time that the normal resting mPAP typically does not exceed 15 mmHg at rest with little impact by age [2]. Over the subsequent decades, this arbitrary choice continued to be controversial but remained essentially unchanged due to the absence of normative data and ethical concerns regarding invasive testing without a clinical indication prevented the collection of such data [16].

The first major breakthrough was a large meta-analysis which reviewed the hemodynamic data of 1187 healthy individuals [17]. In this study, the mPAP in this cohort was found to be 14.0 ± 3.3 mmHg with the upper limit of normal defined as 2 standard deviations above the mean or 20.6 mmHg. This threshold for the upper limit of normal was now established by a scientific approach and was not arbitrarily set in contrast to the original definition. Despite this knowledge, the 2015 ESC/ERS guidelines retained the definition of PH as ≥25 mmHg as the clinical significance of “borderline” elevations in mPAP (20–25 mmHg) had not been adequately studied [9,18]. Appropriately, an update to the hemodynamic definition of PH required data suggesting prognostic implications of the decision [14].

However, data suggesting poorer outcomes in patients with lower levels of mPAP had started to accumulate within a wide range of diseases. This included findings of increased mortality in patients with idiopathic pulmonary fibrosis (IPF), chronic obstructive pulmonary disease (COPD), sickle cell disease, systemic sclerosis, and in a relatively unselected population with concern for PH [19,20,21,22,23]. Further confirmation was provided by two large retrospective database reviews totalling more than 25,000 patients, many whom had left heart disease, revealing an increased hazard ratio for death with mPAP between 19–24 mmHg [24,25]. Further confirmation was provided by pathological data revealing adverse remodeling occurring in patients who had milder elevations in mPAP with systemic sclerosis [26].

This accumulation of normative physiologic data coupled with prognostic data from various conditions led to the adoption of a reduced diagnostic threshold of the mPAP for PH at the 6th WSPH and more recently in the newest ESC/ERS guidelines [1,14].

### 3.2. Pulmonary Arterial Wedge Pressure

By wedging the balloon on the tip of the Swan-Ganz catheter in a mid-sized pulmonary artery a “stop flow” phenomenon is created in the occluded pulmonary artery and the downstream pulmonary capillary bed and pulmonary veins. In the absence of flow, pressure equilibrates across the pulmonary capillary bed so that the pressure in a same-sized pulmonary vein can be estimated by the so called PAWP. A PAWP ≤ 15 mmHg is recommended to distinguish pre-capillary from post-capillary PH [1]. However, it should be noted that a PAWP value of 12 mmHg is generally regarded as the upper limit of normal in healthy individuals [2,27,28]. Recent data have suggested that a PAWP threshold of 12 mmHg provides the highest sensitivity and specificity for distinguishing between pre-capillary and post-capillary PH [29].

However, available data on the best PAWP threshold are contradictory and a higher threshold is recommended for the invasive diagnosis of heart failure by the ESC Heart Failure Association [1,30]. Additionally, almost all randomized controlled trials (RCT) in PAH have utilized an PAWP ≤ 15 mmHg as an inclusion criterion [1]. Consequently, the current ESC/ERS guidelines recommend a PAWP threshold ≤ 15 mmHg is recommended by for the differentiation between pre-capillary and post-capillary PH, while acknowledging the presence of a grey area between 13 and 15 mmHg [1]. This highlights the crucial role of accurately phenotyping patients during the diagnostic evaluation.

### 3.3. Pulmonary Vascular Resistance

PVR has been variably included in the diagnosis of PH over the last several iterations of international guidelines for the management PH [31]. The PVR criterion was first introduced in the proceedings of the 3rd World Symposium in 2003 with a threshold of greater than 3 WU applied only to the definition of PAH but with little discussion on the rationale for inclusion nor the source of this threshold [4]. However, this definition was excluded in the first two iterations of the ESC/ERS guidelines in 2004 and 2009 but was subsequently introduced into the ESC/ERS guidelines in 2015, similarly only applying to the diagnosis of PAH [9,18]. While not specifically addressed in the 2015 ESC/ERS document, the prior World Symposium proceedings outline the rationale for choosing 3 WU instead of 2 WU as patients with a PVR of less than 3 WU are unlikely to have PAH [18].

Similar to the discussion above on changes to the mPAP, the first observations to challenge the prevailing definition were the publication of normative data on PVR among healthy individuals [32]. This systematic review identified that the upper limit of normal for PVR was 2 WU over a large range of ages. However, as the PVR threshold of ≥3 WU was deemed clinically relevant due to its use in other clinical scenarios, such as congenital heart disease and heart transplantation, it was not adopted during the 6th World Symposium [9,10].

Prognostic data were subsequently released to further justify re-examination of the PVR threshold. In a population of systemic sclerosis patients with mildly elevated mPAP (21–24 mmHg), a PVR of ≥2 WU was associated with physiological limitations with reduced walk distances and pulmonary arterial compliance as well as reduced long-term survival [33]. This was shortly followed by a large retrospective review of two large databases where increased all-cause mortality hazard for PVR increased progressively starting around 2 WU, with a clinical significant mortality HR identified at 2.2 WU [34]. This finding was independent of the PAWP, with increased mortality identified in patients with both pre- and post-capillary PH. Pathologic data again confirmed the findings of adverse vascular remodeling in patients with lower PVR beginning approximately at 1.8–2 WU [35].

This evolution in our understanding was thus accepted in the new ESC/ERS guidelines establishing the upper limit of normal and the lowest prognostically relevant PVR threshold of 2 WU within the new definition of pre-capillary PH [1].

### 3.4. Exercise PH

Similar to PVR, exercise PH has been intermittently included in published guidelines since the proceedings of the first world symposium in 1975 [2]. In that document, the authors conclude that the mPAP does not normally exceed 30 mmHg during exercise, but acknowledged that in athletes with high cardiac outputs, pressures have been demonstrated to exceed this value. Despite this, PH on exercise continued to be defined as a mPAP greater than 30 mmHg until the publication of a systematic review of normative data revealed that both age and the level of exercise significantly impacted the mPAP on exercise leading to readings greater than 30 mmHg in otherwise healthy patients [17]. As a result, the ESC/ERS guidelines acknowledged that a definition for PH on exercise, as assessed by RHC, was not supported by the data leading to its removal in 2009 [7].

The definition of exercise PH continued to evolve with a focus on alternative hemodynamic parameters, as it was clear that a pressure threshold alone was not a suitable [36]. The next major development was the publication of a systematic review discussing the flow-dependant changes in exercise hemodynamics [32]. By reviewing 250 patients with exercise hemodynamics, the authors were able to identify linear relationship between the mPAP and CO. Two important observations were that the mPAP/CO slope was positive and that there was a significant increase with age [37]. Specifically, the mean values were 0.8 ± 0.4 mmHg/L/min in patients around age 30, 1.6 ± 0.2 mmHg/L/min around age 50, and 2.4 ± 0.5 mmHg/L/min around age 70 with upper limits of normal of 1.6, 2.1, and 3.3 mmHg/L/min respectively. These data along with other observations established the upper limit of normal for the mPAP/CO relationship around 3 mmHg/L/min [38].

The mPAP/CO slope was subsequently investigated for prognostic relevance similarly to the mPAP and PVR thresholds [39,40,41,42]. The largest study was performed in a group of patients evaluated for unexplained dyspnea [41]. The authors found that an mPAP/CO threshold of 3 mmHg/L/min for exercise PH was associated with a worse cardiovascular (CV) event-free survival regardless of whether there was resting PH. Further, both pre- and post-capillary contributions to the abnormal mPAP/CO slope were independently associated with increased hazard of CV hospitalization or death. In systemic sclerosis patients without manifest PH, exercise PH is a known predictor of disease progression and poor outcomes but further investigation found that an mPAP/CO slope > 3.5 mmHg/L/min identifies those with increased mortality at 10 years despite normal resting hemodynamics [42,43].

Based on these emerging normative and prognostic data, exercise PH was re-introduced into the hemodynamic definitions of PH in the 2022 ESC/ERS updated guidelines [1]. However, while included in the hemodynamic definitions, exercise hemodynamic testing is a technically challenging procedure that may not be available to all PH centres [36].

## 4. Management Strategy

The overall management strategy for patients with pulmonary hypertension remains unchanged compared to the prior guidelines. The guidelines outline general measures, baseline and follow-up risk stratification, and PAH specific therapy use in patients diagnosed with PH. While this article is not focused on the updated treatment paradigm in PAH, one area that warrants discussion are cardiopulmonary comorbidities and their impact on treatment selection.

Over the last few decades, there has been a shift in the demographic of patients diagnosed with idiopathic PAH, specifically patients are being diagnosed older with more comorbidities [44]. Further analysis revealed that, in addition to the classic PAH phenotype (generally young, mostly female patients without comorbidities), two other phenotypes have emerged [45]. One of the phenotypes include elderly, mostly female patients who have risk factors for left heart disease however hemodynamically meet criteria for pre-capillary pulmonary hypertension. The other phenotype is composed primarily of elderly males with comorbidities, a history of smoking, and a low diffusing capacity for carbon monoxide (DLCO). These two phenotypes encompass the majority of patients in registries with idiopathic PAH and have similar recommendations for initial therapy and are referred to in the guidelines as patients with cardiopulmonary comorbidities.

The guidelines differentiate initial treatment selection for those patients without comorbidities (i.e., the classical phenotype) and those with comorbidities [1]. Patients without comorbidities have their initial treatment selection based on risk stratification. Those patients at low- or intermediate-risk are recommended to start with dual upfront combination therapy compared to those at high-risk starting treatment with triple upfront combination therapy including a parenteral prostanoid. Patients who remain intermediate- or high-risk at follow-up are recommended for escalation of therapy.

In contrast, treatment recommendations for patients with cardiopulmonary comorbidities are less clear given the lack of published data as these patients have typically been excluded in modern landmark studies [46]. Data suggest that these patients tend to experience more side effects from PAH therapies leading to discontinuation and have less clinical improvement compared to the classical phenotype [47]. Patients with cardiopulmonary comorbidities are recommended to start treatment with initial monotherapy, regardless of baseline risk stratification. At follow-up, escalation of therapy may be considered on an individualized basis.

## 5. Clinical Implications of the Updated Hemodynamic Definitions

As the hemodynamic definition of PH is the foundation for the diagnosis and subsequent management of patients, changing these definitions has wide reaching implications for both patients and clinicians. A particular tension arises as, currently, no guideline recommended medical therapies exist for patients who meet the new criteria for resting (between mPAP 21–24 mmHg and/or PVR 2–3 WU) or exercise PH [1].

In the previous era of the 2015 ESC/ERS guidelines, patients who met the hemodynamic definition were given a diagnosis of PH. Along with the label of PH, there were serious implications to the patient impacting future quality and quantity of life which were, to some extent, mitigated by the provision of interventions. These included recommendations for general measures such as physical activity, vaccination, psychosocial supports, and management of contributory risk factors in conjunction with the initiation of PAH-specific therapies to directly treat the vasculopathy for those with Group 1 PAH [9].

With the recent update in 2022, there are now a significant number of patients who will receive a life altering diagnosis of PH, resting or with exercise, who would not previously have met criteria [48]. All these patients will benefit from access to multidisciplinary care teams available at PH centres, including specialized nursing, social workers, dieticians, pharmacists, and other allied healthcare workers who serve a critical role in the holistic management of these complex patients. However, the question of PAH therapies in this population has yet to be answered [1]. While early data appear promising, treatment with PAH therapies is reserved only for those patients who were included in prior randomized control trials for these therapeutics, namely those who met the prior hemodynamic definition of an mPAP ≥ 25 mmHg, PAWP of ≤15 mmHg, and PVR of >3 WU [1,49,50,51].

Over the last several years, convincing arguments have been raised against lowering the hemodynamic definitions for PH. The main objection is the risk of overdiagnosis and overtreatment leading to exposure to potentially harmful therapeutics [52]. As the upper limit of normal is defined by two standard deviations, approximately 2.5% of patients will have hemodynamics above these thresholds but be free from disease [31]. This highlights the pivotal role of the PH centre and PH practitioner in identifying patients appropriate for potent, and costly, medical therapies. Even more emphasis will need to be placed on adequate exclusion of alternative causes of PH and ensuring that hemodynamic assessments are performed correctly given the risk of misclassification if performed inadequately [53]. Previously, there existed a gray area between normal hemodynamics and a diagnosis of PAH, specifically those with an mPAP 21–24 mmHg and/or PVR 2–3 WU, which was proposed to help limit overdiagnosis and overtreatment. Now patients with, for instance, Group 2 PH who are misclassified as PAH, perhaps if provocative testing is not performed in a patient with a borderline PAWP, may be at risk of worsening clinical outcomes following exposures to PAH therapies [54]. Indeed, retrospective data already reveals a concerning trend of prescribing PAH therapies off-label and the expected increase in the population of patients diagnosed with PH, particularly with milder disease, may further worsen this trend [55,56]. Special attention is required to carefully evaluate patients at these margins, and they are likely to benefit greatly from specialized evaluation at expert centres, especially given the higher prevalence of cardiorespiratory comorbidities in this group of patients [48]. However, despite increasing awareness of PH among practitioners and the increased availability of screening modalities, namely echocardiography, over the last several decades, patients with a new diagnosis of PAH are still presenting late with hemodynamic abnormalities greatly exceeding these new thresholds [57]. A greater impact of these changes may be felt by patients who are part of screening cohorts, such as those with systemic sclerosis, who are diagnosed earlier with less severe hemodynamic findings [1,58].

Patients meeting the new hemodynamic criteria, but not thresholds for treatment, should be followed regularly given the risk of progression to manifest PH warranting therapy, particularly in groups at high risk of developing PAH [33,42]. The question remains how best to monitor patients for progression as even in systemic sclerosis patients, who are at high risk of developing PAH, the conversion from an mPAP 21–24 mmHg to ≥25 mmHg at 3 years of follow-up occurred in around 25% of patients with only the minority (7%) being diagnosed with PAH [59].

There is a paucity of research in this hemodynamic group of patients and specific attention should be made by the PH community to enrol this group of patients into PH registries and, when available, interventional clinical trials to establish the role of PAH therapies in patients with milder hemodynamic abnormalities.

## 6. Conclusions

The classification and hemodynamic definitions of PH have evolved greatly since the first WHO meeting on chronic cor pulmonale held in Geneva in 1960, in what was only then emerging as a new field of medicine. The 2022 ESC/ERS guidelines for the diagnosis and management of PH outlines the newest and most evidence based hemodynamic definitions for PH that significantly impacts clinical practice. While now supported by firm normative and prognostic data, these changes raise many new questions and identifies exciting new directions for inquiry. Over the years, the hard work and dedication of generations of clinicians and researchers has led, and will continue to lead, to significant advances in our shared understanding of this collection of diseases and improve the lives of patients around the world.

## Figures and Tables

**Table 1:** Current clinical classification of pulmonary hypertension, adapted from [1].

**Group 1: Pulmonary arterial hypertension (PAH)**
1.1 Idiopathic 1.1.1 Non-responders at vasoreactivity testing 1.1.2 Acute responders at vasoreactivity testing 1.2 Heritable 1.3 Associated with drugs and toxins 1.4 Associated with: 1.4.1 Connective tissue disease 1.4.2 HIV infection 1.4.3 Portal hypertension 1.4.4 Congenital heart disease 1.4.5 Schistosomiasis 1.5 PAH with features of venous/capillary (PVOD/PCH) involvement 1.6 Persistent PH of the newborn
**Group 2: PH associated with left heart disease**
2.1 Heart failure: 2.1.1 with preserved ejection fraction 2.1.2 with reduced or mildly reduced ejection fraction 2.2 Valvular heart disease 2.3 Congenital/acquired cardiovascular conditions leading to post-capillary PH
**Group 3: PH associated with lung diseases and/or hypoxia**
3.1 Obstructive lung disease or emphysema 3.2 Restrictive lung disease 3.3 Lung disease with mixed restrictive/obstructive pattern 3.4 Hypoventilation syndromes 3.5 Hypoxia without lung disease (e.g., high altitude) 3.6 Developmental lung disorders
**Group 4: PH associated with pulmonary artery obstructions**
4.1 Chronic Thromboembolic Pulmonary Hypertension (CTEPH) 4.2 Other pulmonary artery obstructions
**Group 5: PH with unclear and/or multifactorial mechanisms**
5.1 Hematological disorders 5.2 Systemic disorders 5.3 Metabolic disorders 5.4 Chronic renal failure with or without hemodialysis 5.5 Pulmonary tumour thrombotic microangiopathy 5.6 Fibrosing mediastinitis

**Table 2:** Current hemodynamic definitions of pulmonary hypertension, adapted from [1].

Hemodynamic Definitions of Pulmonary Hypertension	
Pulmonary Hypertension (PH)	mPAP > 20 mmHg
Pre-capillary PH	mPAP > 20 mmHg PAWP ≤ 15 mmHg PVR > 2 WU
Isolated Post-capillary PH	mPAP > 20 mmHg PAWP > 15 mmHg PVR ≤ 2 WU
Combined pre- and post-capillary PH	mPAP > 20 mmHg PAWP > 15 mmHg PVR > 2 WU
Exercise PH	mPAP/CO slope > 3 mmHg/L/min

mPAP, mean pulmonary artery pressure; PAWP, pulmonary artery wedge pressure; PVR, pulmonary vascular resistance; WU, Wood Units; CO, cardiac output.
